# Genetic Variation of PD-L1 Gene Affects its Expression and Is Related to Clinical Outcome in Epithelial Ovarian Cancer

**DOI:** 10.3389/fonc.2022.763134

**Published:** 2022-05-26

**Authors:** Haiyan Sun, Yan Li, Wengang Si, Tian Hua, Juan Chen, Shan Kang

**Affiliations:** ^1^ Department of Obstetrics and Gynecology, The Fourth Hospital of Hebei Medical University, Shijiazhuang, China; ^2^ Department of Molecular Biology, The Fourth Hospital of Hebei Medical University, Shijiazhuang, China

**Keywords:** epithelial ovarian cancer, PD-L1, polymorphism, risk, prognosis, diagnosis

## Abstract

**Objective:**

This study aims to investigate the effect of polymorphisms of programmed cell death-ligand 1 (PD-L1) on the risk and patient’s outcomes of epithelial ovarian cancer (EOC).

**Methods:**

Totally, 568 patients and 532 healthy women were included. Three polymorphisms in the PD-L1 gene, rs2297136, rs4143815 and rs4742098, were genotyped by the polymerase chain reaction/ligase detection reaction (PCR-LDR). Survival analysis was performed in 234 patients (received primary debulking surgery followed by platinum-based chemotherapy).

**Results:**

Patients with the rs2297136 AG + GG genotypes had shorter progression-free survival (PFS) (hazard ratio (HR)=1.44, 95% CI=1.03-2.01) and overall survival (OS) (HR=1.55, 95% CI=1.06-2.27) than those with the AA genotype. Moreover, the mRNA and protein expression levels of PD-L1 in EOC tissues with the rs2297136 AG + GG genotypes were remarkably higher than those with the AA genotype (P=0.032 and P=0.047, respectively). Survival analysis showed that high expression of PD-L1 mRNA was remarkably associated with worse 10-year PFS (HR=1.55, 95% CI=1.28-1.88) and OS (HR=1.51, 95% CI=1.00-2.28) in EOC patients.

**Conclusions:**

The rs2297136 may not only effectively influence the expression of PD-L1, but also is significantly associated with EOC patients’ outcomes.

## Introduction

Epithelial ovarian cancer (EOC) is one of the most prevalent cancers in women and the leading cause of gynecologic cancer-related death (1). Due to a lack of effective detection strategies, approximately 70% of women are diagnosed at a later stage. The standard treatment for advanced EOC is cytoreductive surgery in combination with platinum-based chemotherapy. Despite much progress in optimizing treatments, the 5-year overall survival (OS) rate is only 30% of women with advanced ovarian cancer (2), and patient prognosis has not improved significantly over the last three decades (1). Fortunately, recent clinical trials of cancer immunotherapy have shown substantial survival benefits of antibodies against either programmed cell death-ligand 1 (PD-L1) or programmed cell death-1 (PD-1) in some types of cancer, including platinum-resistant or refractory EOC (3–5). Therefore, a better understanding of the potential role of PD-L1 in EOC development may guide the clinical use of anti-PD-1/PD-L1 therapy for this malignancy.

Of the human T cell-mediated immune microenvironment, tumor cells have adopted various strategies to evade immune surveillance, including the up-regulation of PD-L1 (6, 7). PD-L1 is the dominant inhibitory ligand of PD-1 on T cells (8, 9). Under normal physiological conditions, the interaction between PD-L1 and PD-1 plays vital roles in maintaining immune tolerance, preventing autoimmune disease and eliminating cancer cells. In the tumor microenvironment, however, cancer cells expressing PD-L1 may evade immune surveillance (10, 11) and thus avoid immune-mediated elimination (12, 13). Although normal tissues have low PD-L1 expression or lack expression completely, most human cancers constitutively express high PD-L1 protein levels (14). It seems logical that over-expression of PD-L1 on tumor cells should be correlated with tumorigenesis and poor prognosis (15–18).

Accumulating evidence suggests that polymorphisms in the 3’-UTR targeted by microRNAs (miRNAs) can alter the expression of target genes and thereby affect the prognosis of cancer (19, 20). Three polymorphisms, rs2297136, rs4143815 and rs4742098, in the miRNA-binding site within the 3’-UTR of PD-L1 have been found to be associated with prognosis in non-small-cell lung cancer (NSCLC), liver cancer, and gastric cancer (21–23). In the current study, we investigated the role of these three polymorphisms in the occurrence risk of EOC and the clinical outcome of patients.

## Materials and Methods

### Study Subjects

In this study, 568 EOC patients and 532 age-matched healthy controls were genetically ethnic Han Chinese population in Hebei Province. All of the subjects were enrolled between January 2007 and March 2018 at the Fourth Hospital of Hebei Medical University. Eligibility inclusion criterion for cases was histologically confirmed primary EOC patients of any age. Patients were excluded if they had other types of cancers and pre-operative radiotherapy or neoadjuvant chemotherapy, a history of human immunodeficiency virus infection, or a history of autoimmune disease such as type I diabetes and systemic lupus erythematosus. Inclusion criterion for control subjects consisted of women without any malignant disease confirmed by surgical exploration, pathological analysis or ultrasound examination. Those patients with any personal or family history of cancer were excluded. All individuals provided written informed consent according to the Declaration of Helsinki. This study was approved by the Ethics Committee of the Fourth Hospital of Hebei Medical University (2018MEC148) and was performed in accordance with the ethical standards stated in the 1964 Declaration of Helsinki and its later amendments or comparable ethical standards.

All EOC patients who underwent primary cytoreductive surgery and received platinum-based chemotherapy were followed up between January 2007 and March 2018. The International Federation of Gynecology and Obstetrics (FIGO) staging revealed that 113 patients were in stage I, 51 patients in stage II, 371 patients in stage III, and 33 patients in stage IV. Patients with FIGO stage IB-IIC disease received 3-6 cycles of chemotherapy, while patients with FIGO stage IIIA-IV disease received 6-8 cycles. In this group of patients, 234 were followed up for more than 5 years. Standard surveillance consisted of serial physical examinations, serum CA-125 testing, and CT scanning. All clinical data were recorded and assessed without knowledge of the genotype status.

### DNA Extraction and Genotyping

Whole-blood specimens (5ml) were collected from each subject in EDTA-coated tubes and stored at 4°C. Genomic DNA was isolated from blood samples by standard salting-out method (24) within a week. The DNA samples were dissolved in TE buffer and stored at -20°C. The genotypes of three PD-L1 polymorphisms were determined by Shanghai Generay Biotech Co., Ltd., using the polymerase chain reaction/ligase detection reaction (PCR-LDR) method. The process was referred to previous report (25).

### RNA Extraction and Quantitative Real-Time Reverse Transcriptase-PCR

During the primary surgery, EOC tissues were collected from patients who received no anti-tumor therapy before surgery. Total RNA was isolated from EOC tissue specimens using TRIzol reagent following manufacturer’s instructions. The RNA was then reverse-transcribed into cDNA using the First Strand cDNA synthesis kit. For qPCR, GAPDH was used as an internal control, and the primers for PD-L1 and control were designed by Sangon Biotech Co., Ltd. Primer sequences are listed in **Table 1**. RT-qPCR was performed using SYBR-Green II Premix. The relative expression of PD-L1 mRNA in each group was calculated using the 2^−ΔΔCt^ method. Each reaction was repeated three times.

**Table 1:** Primer sequences of *PD-L1* gene polymorphisms.

SNP	Primer	Primer sequence	PCR product length
rs2297136(A/G)	Forward	ATCTTTCTTCATTCTCCTCCTCTG	164bp
	Reverse	ATCTTCAAGCAGGGATTCTCAA	
rs4143815(C/G)	Forward	AGGAAGACGGGTTGAGAATC	176bp
	Reverse	GACAAGAAGACCTCACAGACTC	
rs4742098(G/A)	Forward	GCATAGGCAGAGATGATACCT	165bp
	Reverse	TCCACTGGGATGTTAAACTGAA	

SNP: single nucleotide polymorphism; PCR: polymerase chain reaction.

### Immunohistochemistry

49 tissue samples for IHC staining, 15 for the AA genotype analysis, 19 for the AG genotype analysis and 15 for the GG genotype analysis, were selected from 568 EOC patients. IHC staining for PD-L1 protein was performed on 4-µm-thick sections by the avidin–biotin peroxidase complex method. Consecutively, tissue sections were dewaxed in xylene and dehydrated in graded ethanol. After blocking endogenous peroxidase activity and non-specific antibody binding, sections were incubated with primary antibody (rabbit polyclonal anti-PD-L1 antibody, Abcam, ab205921, Cambridge, UK; dilution 1:100) overnight at 4°C, and then with biotinylated secondary antibody and avidin-biotin-peroxidase complex. After washing the slides in PBS, they were incubated in DAB (brown) and counterstained with haematoxylin (blue). In the negative control, primary antibody was replaced by PBS. Without knowledge of the clinical data for each patient, 2 observers independently evaluated and interpreted the results of IHC staining (membrane staining). Agreement was determined by intraclass correlation coefficient for continuous variables, and Fleiss’ kappa (κ) for categorical variables. The immune cells were avoided. IHC staining was evaluated by a previously reported scoring method (26). The score was established corresponding to the sum of: (1) the percentage of positive cells (0, 0% positive cells; 1, < 25% positive cells; 2, 26–50% positive cells; 3, > 50% positive cells); and (2) the staining intensity (0, negative; 1, weak; 2, moderate; 3, high). The sum for the assigned values (the positive cell percentage and the staining intensity) was 6 or less than 6. Scores between 0 and 2 was regarded as negative, 3 and 4 as weakly positive, and 5 and 6 as strongly positive.

### Kaplan-Meier Plotter Database Analysis

The prognostic value of PD-L1 mRNA levels in patients with EOC was determined using the “K-M plotter” database, integrated gene expression data and survival information of 1,816 EOC patients. In this study, PFS and OS of EOC patients for over 10 years were evaluated using the K-M plotter. The exclude outlier arrays were selected as the array quality control.

### Statistical Analysis

Statistical analysis was conducted using the SPSS v21.0, and a probability level of 5% was considered to indicate significance. Hardy-Weinberg equilibrium analysis was performed to compare the observed and expected genotype frequencies using the χ2 test in the control group. Differences in genotype/allele distribution in the cases and controls were compared using the χ2 test with Bonferroni’s correction. Unconditional logistic regression models were used to calculate odds ratios (ORs) and 95% confidence intervals (CIs). Nonparametric unpaired Mann-Whitney U test was used to compare mRNA expression between groups. Pearson Chi-square tests were conducted to compare PD-L1 protein expression in tumor tissues. Survival analysis was carried out using the K-M method with log-rank test. Multivariate analysis was performed using Cox proportional hazards regression models. A total of 5 factors were included in the multivariate analysis, including age, stage, tumor grade, pathology and residual tumor.

## Results

### Demographic Profile

The median age was 54 years (ranged from 20 to 77) for the patients and 54 years (ranged from 20 to 79) for the controls. There was no significant difference in age distribution between the patients and controls (P>0.05). The genotypes of the three PD-L1 polymorphisms did not deviate significantly from HWE in the controls. **Table 2** showed clinical characteristics of patients stratified by the three polymorphisms.

**Table 2:** The association between *PD-L1* polymorphisms and clinical characteristics of EOC patients.

Group	rs2297136 (A>G) n (%)		P	rs4143815 (C>G) n (%)		P	rs4742098 (G>A) n (%)		P
	AA	AG + GG		CC	CG + GG		GG	GA + AA	
**Age**									
**<50**	127 (72.2)	49 (27.8)	0.068	64 (36.4)	112 (63.6)	0.850	53 (30.1)	123 (69.9)	0.845
**≥50**	251 (64.0)	141 (36.0)		139 (35.5)	253 (64.5)		122 (31.1)	270 (68.9)	
**Stage**									
**I**	78 (68.1)	35 (30.9)	0.706	44 (38.9)	69 (61.1)	0.238	37 (32.7)	76 (67.3)	0.152
**II**	35 (68.6)	16 (31.4)		13 (25.5)	38 (74.5)		10 (19.6)	41 (80.4)	
**III**	241 (64.9)	130 (35.1)		131 (35.3)	240 (64.7)		114 (30.7)	257 (69.3)	
**IV**	24 (72.7)	9 (27.3)		15 (45.5)	18 (54.5)		14 (42.4)	19 (57.6)	
**Tumor grade**									
**1**	199 (70.1)	85 (29.9)	0.200	104 (36.6)	180 (63.4)	0.909	83 (29.2)	201 (70.8)	0.548
**2**	165 (63.2)	96 (36.8)		91 (34.9)	170 (65.1)		83 (31.8)	178 (68.2)	
**3**	14 (60.9)	9 (39.1)		8 (34.8)	15(65.2)		9 (39.1)	14 (60.9)	
**Pathology**									
HGSOC	256 (67.7)	122 (32.3)	0.355	138 (36.5)	240 (63.5)	0.521	122 (32.3)	256 (67.7)	0.139
**Endometrioid**	70 (62.5)	42 (37.5)		34 (30.3)	78 (69.7)		25 (22.3)	87 (77.7)	
**Mucinous**	17 (56.7)	13 (43.3)		11 (36.7)	19 (63.3)		12 (40.0)	18 (60.0)	
**Others**	35 (72.9)	13 (27.1)		20 (41.7)	28 (58.3)		16 (33.3)	32 (66.7)	
**Residual tumor**									
**R0**	169 (70.1)	72 (29.9)	0.289	84 (34.9)	157 (65.1)	0.147	69 (28.5)	173 (71.5)	0.191
**≤1 cm**	55 (63.9)	31 (36.1)		24 (27.6)	63 (72.4)		22 (25.6)	64 (74.4)	
**>1cm**	153 (63.7)	87 (36.3)		94 (39.2)	146 (60.8)		83 (34.6)	157 (65.4)	

HGSOC, high grade serous ovarian cancer.

### Associations of the Three PD-L1 Polymorphisms With the Risk of Developing EOC

We analyzed the genotype and allele frequencies of the three PD-L1 polymorphisms. As showed in **Table 3**, there were no significant differences in the genotype and allele frequencies of these polymorphisms between the cases and controls. It is indicated that the three polymorphisms of PD-L1 were not related to the risk of EOC in northern Chinese population.

**Table 3:** Association between the three polymorphisms and the risk of EOC patients.

Polymorphism	Genotype/allele	Controls (%)	Cases (%)	P	OR	95%CI
rs2297136	AA	358 (67.3)	378 (66.5)	0.793	1.00	1-1
	AG + GG	174 (32.7)	190 (33.5)		1.034	0.804-1.330
	A	875 (82.2)	931 (82.0)	0.863	1.00	1-1
	G	189 (17.8)	205 (18.0)		1.019	0.820-1.26
rs4143815	CC	208 (39.1)	203 (35.7)	0.250	1.00	1-1
	CG + GG	324 (60.9)	365 (64.3)		1.323	0.904-1.474
	C	656 (61.7)	692 (60.9)	0.722	1.00	1-1
	G	408 (38.3)	444 (39.1)		1.032	0.869-1.225
rs4742098	GG	160 (30.1)	175 (30.8)	0.791	1.00	1-1
	GA + AA	372 (69.9)	393 (69.2)		0.966	0.747-1.249
	G	599 (56.3)	646 (56.9)	0.788	1.00	1-1
	A	465 (43.7)	490 (43.1)		0.977	0.825-1.157

OR, odds ratio.

### Association Between PD-L1 Polymorphisms and the Clinical Outcome of EOC Patients

**Figure 1:**
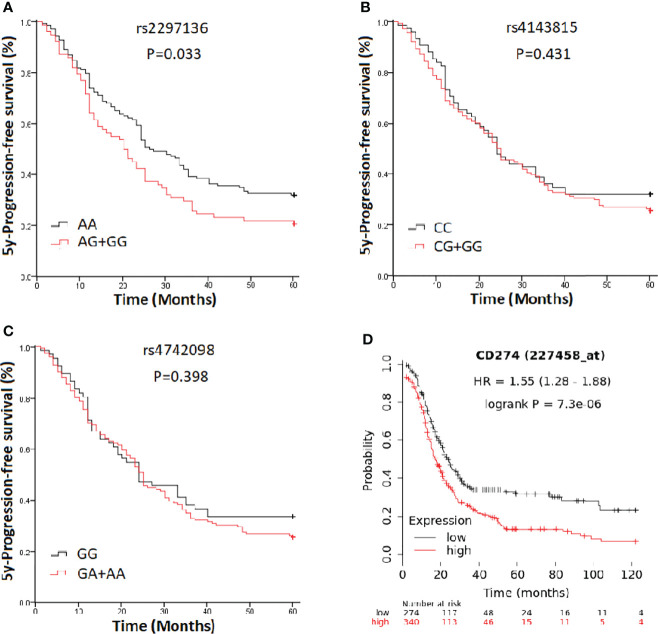
Kaplan-Meier estimate of progression-free survival (PFS) in epithelial ovarian cancer patients categorized by PD-L1 polymorphisms. **(A)** rs2297136; **(B)** rs4143815; **(C)** rs4742098; **(D)** PD-L1expression.

**Figure 2:**
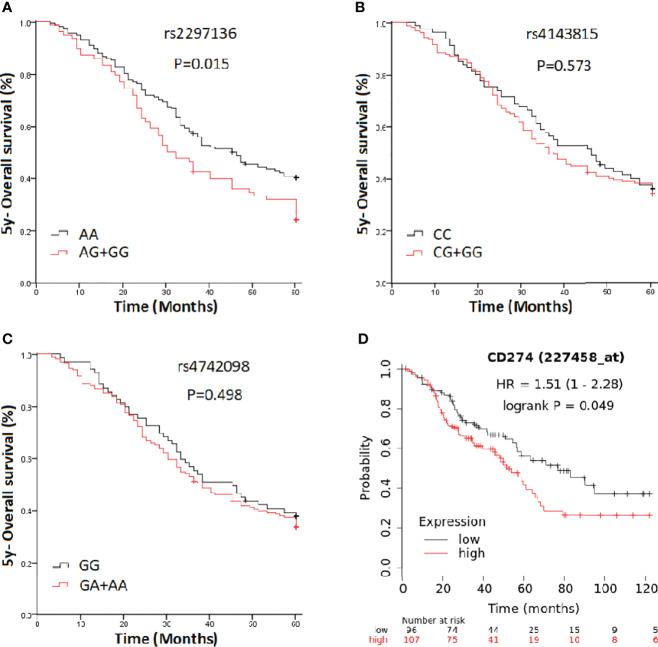
Kaplan-Meier estimates of overall survival (OS) in epithelial ovarian cancer patients categorized by PD-L1 polymorphisms. **(A)** rs2297136; **(B)** rs4143815; **(C)** rs4742098; **(D)** PD-L1expression.

In a clinical follow-up study, the median PFS of EOC patients carrying the AA and AG + GG genotypes of PD-L1 rs2297136 was 26 and 20 months, respectively. The median OS of these patients was 45.00 and 30.00 months, respectively. K-M plots showed that patients carrying the AG + GG genotypes had a significantly decreased PFS (P=0.033) (**Figure 1A**) and OS (P=0.015) (**Figure 2A**) compared with those carrying the AA genotype. Further, after adjusting for prognostic factors including age, stage, pathology (high grade serous ovarian cancer, endometrioid, mucinous and others) and tumor residual size (R0, ≤1 cm and >1cm), patients with the AG + GG genotypes had an increased risk of disease progression (HR = 1.44, 95%CI = 1.03–2.01, P=0.033) and death (HR = 1.55, 95%CI = 1.06–2.27, P=0.025) compared with those carrying the AA genotype (**Table 4**). However, the rs4143815 and rs4742098 polymorphisms were not associated with the prognosis of patients with EOC (**Table 4**).

**Table 4:** Univariate analysis of prognostic factors for 5-year progression-free survival and overall survival of EOC patients.

Group	Recurrence		HR (95%CI)	P	PFS	Survival		HR (95%CI)	P	OS
	No n (%)	Yes n (%)				Alive n (%)	Dead n (%)			
**Age**										
**<50**	30 (39.5)	46 (60.5)	1-1		38	37 (48.7)	39 (51.3)	1-1		45
**≥50**	30 (19.0)	128 (81.0)	1.331 (0.899-1.969)	0.153	26	45 (28.5)	113 (71.5)	1.439 (0.969-2.135)	0.071	36
**Stage**										
**I/II**	36 (60.0)	24 (40.0)	1-1		48	43 (71.7)	17(28.3)	1-1		51
**III/IV**	24 (13.9)	149 (86.1)	1.773 (0.931-3.376)	0.081	24	39 (22.5)	134 (77.5)	2.080 (1.073-4.033)	0.030	35
**Pathology**										
HGSOC	33 (19.9)	133 (80.1)	1-1		23	51 (30.7)	115 (69.3)	1-1		34
**Others**	27 (39.7)	41(60.3)	0.861 (0.586-1.266)	0.446	36	31 (45.6)	37 (54.4)	0.997 (0.677-1.468)	0.989	53
**Residual tumor**										
**R0**	53 (53.5)	46 (46.5)	1-1		45	62 (62.6)	37 (37.4)	1-1		50
**≤1 cm**	5 (13.2)	33 (86.8)	2.730 (1.500-4.969)	0.001	25	10 (26.3)	28 (73.7)	1.998 (1.109-3.599)	0.021	36
**>1cm**	2 (2.1)	94 (97.9)	5.121 (2.985-8.785)	<0.001	16	10 (10.4)	86 (89.6)	3.334 (1.993-5.577)	<0.001	29
**rs2297136**										
**AA**	44 (28.2)	112 (71.8)	1-1		26	63 (40.4)	93 (59.6)	1-1		45
**AG+GG**	16 (20.5)	62 (79.5)	1.438 (1.030-2.008)	0.033	20	27 (39.1)	42 (60.9)	1.549 (1.057-2.270)	0.025	30
**rs4143815**										
**CC**	24 (30.0)	56 (70.0)	1-1		24	29 (36.2)	51 (63.8)	1-1		46
**CG+GG**	36 (23.4)	118 (76.6)	1.417 (0.685-2.933)	0.348	25	54 (43.5)	70 (56.5)	1.265 (0.662-2.417)	0.476	36
**rs4742098**										
**GG**	22 (31.9)	47 (68.1)	1-1		24	26 (37.7)	43 (62.3)	1-1		45
**GA+AA**	38 (23.0)	127 (77.0)	0.901 (0.426-1.904)	0.785	25	52 (41.6)	73 (58.4)	0.853 (0.435-1.675)	0.645	38

HR, hazard ratio; PFS, progression-free survival; OS, overall survival; HGSOC, high grade serous ovarian cancer.

### Rs2297136 Genotype-Dependent Expression of PD-L1 mRNA and Protein in EOC

**Figure 3:**
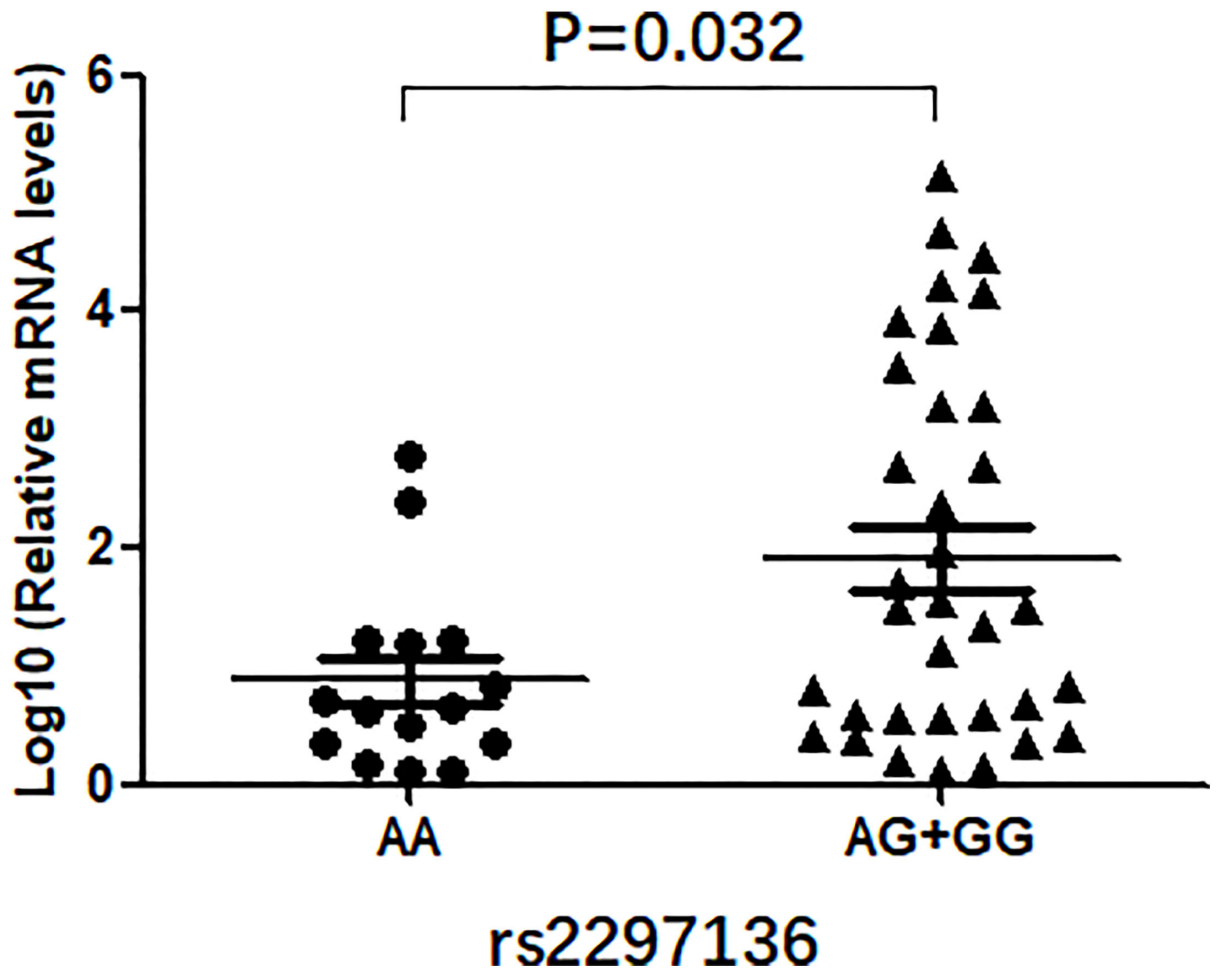
Relative expressions of PD-L1 mRNA in AA and AG + GG genotypes of rs2297136.

**Figure 4:**
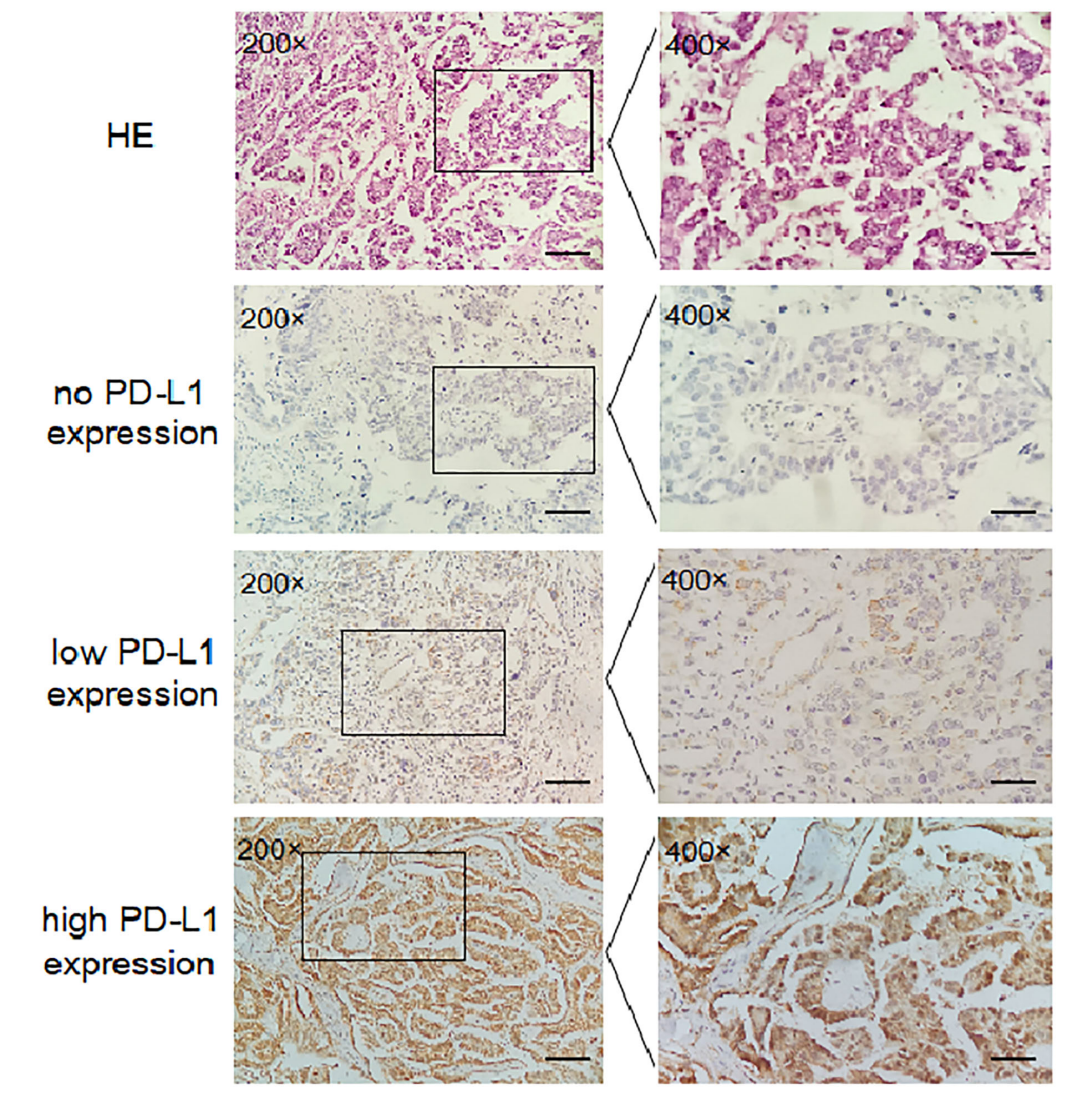
Expression of PD-L1 in EOC tissues detected by IHC (200× and 400×). HE; no PD-L1 expression; low PD-L1 expression; high PD-L1 expression.

In this study, we evaluated the mRNA levels of PD-L1 in EOC tissues from patients carrying 3 genotypes of rs2297136, rs4143815 and rs4742098. The RT-qPCR results showed that the mRNA expression levels of PD-L1 in tumor tissues from patients with the AG + GG genotypes of rs2297136 were remarkably higher than those from patients with the AA genotype (**Figure 3**, P=0.032). This result was further validated by protein expression using IHC staining analysis (**Figure 4**) (**Table 5**). Spearman’s correlation analysis showed that there was a significant positive correlation between PD-L1 mRNA and protein expression in EOC tissues from patients carrying different genotypes of rs2297136 (r=0.411, P=0.009) (**Table 6**). These data implied that rs2297136 polymorphism could be involved in regulation of the expression of PD-L1 mRNA and protein in EOC patients. However, no significant statistical difference was found in EOC patients with genotypes of rs4143815 and rs4742098 (data was not shown).

**Table 5:** Effects of rs2297136 on the expression of PD-L1 protein in tumor tissue of EOC patients.

Genotype/expression	No/Low expression (%)	High expression (%)	P
AA	11 (73.3)	4 (26.7)	1
AG	11 (57.9)	8 (42.1)	0.010
GG	3 (20.0)	12 (80.0)	
AG + GG	14 (41.2)	20 (58.8)	0.038

**Table 6:** Spearman’s correlation analysis between *PD-L1* mRNA and protein expression in EOC patients carrying different genotypes of rs2297136.

PD-L1 mRNA/protein	No/Low expression (%)	High expression (%)	r	P
Low expression	17 (48.0)	8 (32.0)	0.347	0.015
High expression	8 (33.3)	16 (66.7)		

### Prognostic Value of PD-L1 mRNA Expression in EOC Patients

K-M Plotter database [http://kmplot.com/analysis] was used for prognostic analysis. PD-L1 mRNA high expression was associated with significantly shorter PFS (HR=1.55, 95% CI=1.28-1.88; P=7.3×10-6) (**Figure 1D**) for all EOC patients, and OS in grade I, II EOC patients (HR=1.51, 95% CI=1.00-2.28; P=0.049) (**Figure 2D**) followed for 10 years. The median PFS of EOC patients with PD-L1 mRNA high expression and low expression was 14.37 and 20.00 months, respectively. The median OS of these patients was 50.00 and 75.03 months, respectively.

## Discussion

In this paper, we investigated the effect of three polymorphisms, rs2297136, rs4143815 and rs4742098 in the 3’-UTR of PD-L1 on the risk of EOC development and the clinical outcomes of patients in northern China. The results showed that rs2297136 was significantly associated with EOC patient outcomes by changing the expression of PD-L1. Compared to patients with AA genotype, patients carrying the rs2297136 AG + GG genotypes had poorer PFS and OS. To further validate the result above, we analyzed the correlations of PD-L1 mRNA expression with the clinical outcome of EOC patients using the K-M plotter database. The results also indicated that the over-expression of PD-L1 mRNA was significantly correlated with shorter duration of PFS and OS as compared to low-expression of PD-L1 mRNA in EOC patients. However, there was no statistical significance between rs2297136, rs4143815 and rs4742098 polymorphisms and the risk of EOC. To the best of our knowledge, this is the first study to associate the PD-L1 rs2297136 and rs4742098 with the risk of EOC development and with patients’ clinical outcomes.

The rs2297136 is an A-to-G mutation in the 3’-UTR of PD-L1. It is shown that rs2297136 could affect PD-L1 expression by modulating the miRNA-mRNA interaction (26, 27). In this study, our results demonstrated that PD-L1 expression in rs2297136 AG + GG genotypes carriers in EOC patients was higher than that in AA genotype, suggesting that the AG + GG genotype may be related to up-regulation of PD-L1 expression. Some studies have focused on the relationship between the rs2297136 and cancer risk. For example, rs2297136 has been found not related to the risk of gastric cancer (23). Nevertheless, it is (27) found that the AG genotype of rs2297136 was associated with an increased risk of NSCLC. Xie et al. (21) suggested that the rs2297136 TT genotype (variant genotype) significantly increased the risk of hepatocellular carcinoma and decreased the OS of patients in the Chinese Han population. It is worth noting that the frequency of genotypes reported by Xie et al. (21) differs from that reported in the National Center for Biotechnology Information Search database. In this study, although the rs2297136 is not related to the risk of EOC, it may be correlated with a poor prognosis of EOC patients. Compared with the AA genotype, the rs2297136 AG + GG genotypes might significantly decrease the 5-year PFS and OS of EOC patients. Moreover, RT-qPCR and IHC staining confirmed that PD-L1 expression in rs2297136 AG + GG genotypes carriers in EOC patients was higher than that in AA genotype, supporting the speculation that the rs2297136 AG + GG genotypes could lead to significantly shorter PFS and OS in EOC patients than the AA genotype by up-regulating PD-L1 expression. Until now, there have been inconsistent results of PD-L1 expression in EOC tissues on the prognosis of patients with EOC. Using K-M plotter database analysis, the result revealed that over expression of PD-L1 mRNA was significantly associated with worse 10-year PFS and OS in EOC patients, further confirming our above speculation. The expression and function of PD-L1/PD-1 pathway in the human cancer microenvironment (27, 28) was closely associated with tumor immune response, and the PD-L1 protein expression in tumor cells may predict responses to immune checkpoint inhibitors (29, 30). In the Phase II KEYNOTE-100 study, the largest study to date of single-agent immunity checkpoint for recurrent ovarian cancer, higher PD-L1 expression on tumor cells, lymphocytes, and macrophages correlated with higher pembrolizumab monotherapy (anti-PD-L1) response (31). In this study, the rs2297136 AG + GG genotypes were associated with an up-regulated expression of PD-L1, indicating an unfavorable survival outcome of EOC patients with traditional mainstay therapy. Another important implication of the present study is that EOC patients with the rs2297136 AG + GG genotypes may have a good response to the novel immunotherapy targeting PD-L1/PD-1, particularly those for which no effective therapy is currently available.

The rs4143815C/G and rs4742098G/A polymorphisms also located in the miRNA binding region of the 3’-UTR of PD-L1. Dual-luciferase reporter assays showed that the expression of the rs4742098 A allele was significantly reduced due to inhibition by miR-138 (32). For the rs4143815 polymorphism, the C allele may cause the loss of miR-570 binding sites and increase the expression of PD-L1 (22, 31). The association between rs4143815 and cancer risk has been extensively studied. Two meta-analyses suggested that rs4143815 might confer an increased risk of gastric cancer, bladder cancer and hepatocellular carcinoma (27, 33). There is only one study on the correlation of rs4143815 with ovarian cancer, and the results indicated that individuals carrying the rs4143815 GG genotype may have a significantly increased risk of EOC development and that patients with the CG+GG genotype have a poor clinical outcome (16). However, our study did not find that rs4143815 was associated with genetic susceptibility to EOC or clinical patient outcomes. We consider that differences in genotype frequencies in the two studies may underlie these inconsistent results. In addition, studies on the relationship between rs4143815 and the clinical prognosis of cancer patients have mainly focused on NSCLC, but the results are conflicting. Two studies suggested that NSCLC patients with the rs4143815 CC genotype may have a better prognosis (22, 34), but two other studies did not show an association of this polymorphism with the clinical prognosis of NSCLC patients (32, 35). To date, there have been limited studies on the association of rs4742098 with cancer. It is reported that the AG genotype of rs4742098 conferred an increased risk of NSCLC compared with the AA genotype. In our study, we found no association between this polymorphism and the risk of EOC development or the clinical outcome of patients. Studies of other types of tumors are needed to provide additional evidence.

In conclusion, this study showed that the rs2297136 GG genotype was associated with an up-regulated PD-L1 expression and a poor prognosis of EOC among women from Northern China. It would be interesting to determine the underlying molecular mechanisms of the rs2297136 genotype-mediated regulation of PD-L1 expression. However, there are limitations to our study. Firstly, scoring system that reflects established systems in the clinical literature, such as the tumor proportion score (TPS) and combined positive score (CPS) should be applied in the further study. Secondly, further mechanism studies are still necessary to strengthen our conclusions.

## Data Availability Statement

The original contributions presented in the study are included in the article/supplementary material. Further inquiries can be directed to the corresponding author.

## Ethics Statement

The studies involving human participants were reviewed and approved by This study was approved by the Ethics Committee of the Fourth Hospital of Hebei Medical University (2018MEC148) and was performed in accordance with the ethical standards stated in the 1964 Declaration of Helsinki and its later amendments or comparable ethical standards. The patients/participants provided their written informed consent to participate in this study.

## Author Contributions

The contribution of the individual authors to the manuscript included SK and YL designed the study and applied for Research Ethics Board approval. WS and TH recruited the patients and collected the data. WS and HS analyzed the data and prepared draft figures and tables. HS prepared the manuscript draft with important intellectual input from SK and YL. All authors approved the final manuscript. WS, HS and TH had complete access to the study data.

## Funding

This work was supported by the Scientific Foundation of Hebei Province [grant number: 182777171], and the Scientific Research Fund of Hebei Provincial Health and Family Planning Commission [grant number: 20200099], and the Natural Science Foundation of Hebei Province [grant number: H2020206385].

## Conflict of Interest

The authors declare that the research was conducted in the absence of any commercial or financial relationships that could be construed as a potential conflict of interest.

## Publisher’s Note

All claims expressed in this article are solely those of the authors and do not necessarily represent those of their affiliated organizations, or those of the publisher, the editors and the reviewers. Any product that may be evaluated in this article, or claim that may be made by its manufacturer, is not guaranteed or endorsed by the publisher.
